# COPING STRATEGIES AMONG HISPANIC AND LATINX DEMENTIA CAREGIVERS

**DOI:** 10.1093/geroni/igae098.3613

**Published:** 2024-12-31

**Authors:** Nury Rodriguez Colmenares, Danny Wang, Loreli Alvarez, Katie Estes, Natashia Bibriescas, Frank Puga

**Affiliations:** University of Alabama at Birmingham, Birmingham, Alabama, United States; The Pennsylvania State University, University Park, Pennsylvania, United States; University of Alabama at Birmingham, Birmingham, Alabama, United States; University of Alabama at Birmingham, Birmingham, Alabama, United States; The University of Texas Austin, Austin, Texas, United States; University of Alabama at Birmingham, Birmingham, Alabama, United States

## Abstract

The objective of this study was to examine coping strategies employed by Hispanic and Latinx (H&L) family caregivers (FCGs) of people living with dementia (PLWD) and associations with sociodemographic characteristics. A nonparametric analysis was used to analyze data from an ongoing study on the daily experiences of H&L dementia caregivers, entitled Nuestros Días. A total of 52 H&L FCGs were assessed using Brief-COPE. Most H&L FCGs identified as female (92.2%), with a mean age of 56 years (SD = 8.6), were adult children caring for biological parent (59.6%), and provided care for 3 or more years (61.5%). Most PLWD were female (68%) with a mean age of 78 years (SD= 8.6). Overall coping strategies most frequently utilized by H&L FCGs included acceptance (M= 3.14, SD= 1), religion (M= 2.98, SD= 1), positive reframing (M=2.91, SD=0.9), and planning (M=2.8, SD=1). Conversely, substance use (M=1.13, SD=0.3), behavioral disengagement (M=1.39, SD=0.72), denial (M=1.44, SD=0.8), and humor (M=1.47, SD=0.8) were the least utilized by H&L FCGs. The use of acceptance as a coping strategy was found to have a significant association with employment status with working caregivers more frequently endorsing acceptance as a coping strategy (p=.04; Cramer’s V=.409). These findings suggest that H&L caregivers utilize specific coping strategies and that sociodemographic factors, such as employment, may influence coping strategies use d. Additional studies are needed to examine the relationship between additional contextual factors (e.g., caregiving-related stressors and burden) and types of coping strategies used by H&L FCGs to inform ecologically valid and cultural interventions.

